# Interplay between gut microbiota and the master iron regulator, hepcidin, in the pathogenesis of liver fibrosis

**DOI:** 10.1093/femspd/ftae005

**Published:** 2024-03-30

**Authors:** Sara Ahmadi Badi, Ahmad Bereimipour, Pejman Rohani, Shohreh Khatami, Seyed Davar Siadat

**Affiliations:** Biochemistry Department, Pasteur Institute of Iran, Tehran, 1963737611, Iran; Pediatric Gastroenterology and Hepatology Research Center, Pediatrics Center of Excellence, Children's Medical Center, Tehran University of Medical Science, Tehran, 1416753955, Iran; Department of Biological Sciences and BioDiscovery Institute, University of North Texas, Denton, TX 76203, USA; Pediatric Gastroenterology and Hepatology Research Center, Pediatrics Center of Excellence, Children's Medical Center, Tehran University of Medical Science, Tehran, 1416753955, Iran; Biochemistry Department, Pasteur Institute of Iran, Tehran, 1963737611, Iran; Microbiology Research Center, Pasteur Institute of Iran, Tehran, 1963737611, Iran; Department of Mycobacteriology and Pulmonary Research, Pasteur Institute of Iran, Tehran,1963737611, Iran

**Keywords:** liver Fibrosis, hepcidin, iron homeostasis, Gut microbiota

## Abstract

**Introduction:** There is a proven role for hepcidin and the composition of gut microbiota and its derivatives in the pathophysiology of liver fibrosis. **Area covered:** This review focuses on the literature search regarding the effect of hepcidin and gut microbiota on regulating liver physiology. We presented the regulating mechanisms of hepcidin expression and discussed the possible interaction between gut microbiota and hepcidin regulation. Furthermore, we investigated the importance of the hepcidin gene in biological processes and bacterial interactions using bioinformatics analysis. **Expert Opinion:** One of the main features of liver fibrosis is iron accumulation in hepatic cells, including hepatocytes. This accumulation can induce an oxidative stress response, inflammation, and activation of hepatic stellate cells. Hepcidin is a crucial regulator of iron by targeting ferroportin expressed on hepatocytes, macrophages, and enterocytes. Various stimuli, such as iron load and inflammatory signals, control hepcidin regulation. Furthermore, a bidirectional relationship exists between iron and the composition and metabolic activity of gut microbiota. We explored the potential of gut microbiota to influence hepcidin expression and potentially manage liver fibrosis, as the regulation of iron metabolism plays a crucial role in this context.

## Teaser

The authors discuss a new axis for hepcidin in liver fibrosis.

## Article highlights

- Hepcidin is a hepatokine derived mainly from hepatocytes that serves as a master regulator of iron. It controls both systemic and intracellular iron levels.
- Gut microbiota affects liver function by modulation of the gut-liver axis.
- Iron levels can influence gut microbiota composition and metabolic activity.
- Iron availability may be a pivotal regulator in preserving the symbiotic relationship between gut microbiota and the host.
- Iron storage and expression of intestinal iron absorption/exporter-mediated proteins, such as DMT1, DCYTB, and FPN, could be controlled by gut microbiota.
- Gut microbiota may modulate hepatocyte hepcidin gene expression, especially via direct and macrophage-mediating effects
- There is a possible interaction between gut microbiota and hepcidin expression in determining normal liver function and pathological fibrosis.

## Introduction

The liver is a heterogeneous tissue composed of various cells such as hepatocytes, hepatic stellate cells (HSCs), resident macrophages (Kupffer cells), infiltrated immune cells, and Liver Sinusoidal Endothelial Cells (LSECs). These cells undergo morphological and phonotypical changes during liver injury to participate in the liver fibrosis process by over-production of Extracellular Matrix (ECM). Also, the crosstalk between the putative cells is disrupted to induce profibrotic responses under liver injury. The normal function and structure of the liver depend on the desired functioning of cells, especially hepatocytes, HSCs, Kupffer cells, and their cross-talk, to maintain liver structure and restore liver damage. Under pathophysiological condition, the normal function of liver is disrupted and could be followed by pathological fibrosis, cirrhosis and hepatocellular carcinoma (HCC) (Aydin and Akcali 2018). It has been reported the important role of hepcidin, as a systemic iron regulator, and gut microbiota on the onset and development of liver fibrosis which we discussed their molecular signaling and also possible interactions here.

### Liver fibrosis and Hepcidin

One of the primary mechanisms for repairing liver injury is hepatic fibrosis mediated by activated HSCs and ECM proteins (Lu et al. 2016, Kisseleva and Brenner 2021). HSCs are resident hepatic cells in quiescent phenotype, which transdifferentiate to activated cells in myofibroblast-like phenotype after exposure to liver injury. Also, myofibroblast cells could originate from portal fibroblasts and fibroblasts derived from bone marrow. Activated HSCs are the primary fibrogenic cells that restore injured tissue by producing crosslinked collagen types I and III (Tsuchida and Friedman 2017). This prosses is a normal hepatic fibrosis that could be reversed by apoptosis of activated HSCs, reversed transdifferentiation to inactivated form, and activity of Matrix Metalloproteinases (MMPs) after elimination of liver injury (Fallowfield et al. 2007). In contrast, pathogenic liver fibrosis results from chronic hepatotoxic damages (such as hepatitis B virus (HBV), hepatitis C virus (HCV), alcoholic liver disease (ALD), and non-alcoholic steatohepatitis (NASH)) or bile flow obstruction, and cholestatic injury (such as biliary cholangitis, primary sclerosing cholangitis (PSC) and biliary atresia) (Lu et al. 2016, Weiskirchen et al. 2018).

It has been demonstrated the main features of pathogenic fibrosis and cirrhosis include disruption of epithelial and endothelial barriers and inflammatory mediators overproduction, such as TGF-β, by macrophages and infiltrated bone marrow-derived immune cells and accumulation of ECM proteins (Bataller and Brenner 2005). Also, TGF-β, mainly produced by bone marrow-derived macrophages, is the most potent fibrogenic cytokine inducing ECM production through activating the main transcriptional factors, Sma and Mother Against Decapentaplegic (SMADs) (Khedr and Khedr). Unlike bone marrow-derived macrophages, hepatic resident macrophages (Kupffer cells) dually act in liver fibrosis pathology due to anti-inflammatory activity and production of MMPs such as MMP2 and MMP9 to resolve ECM in reversible fibrosis. Therefore, inflammation has been considered as critical inducer of HSCs activation and fibrotic scar formation (Ramachandran et al. 2012, Tsuchida and Friedman 2017).

There is a crucial interaction between iron homeostasis and oxidative stress due to the favor of Fe^+2^ in the chemistry of the Fenton-Haber-Wiess reaction. This reaction yields extremely reactive oxygen species (ROS) from hydrogen peroxide by Fe^+2^ oxidation (Sousa et al. 2020). In a physiologic state, the deleterious effects of ROS which is released normally during cellular metabolisms, are counteracted by the activity of the cellular anti-oxidant system. Furthermore, to inhibit the toxic potential of iron (Fe^+2^) to induce oxidative stress by the Fenton reaction, it is tightly bound to ferritin and transferrin in the intracellular and extracellular spaces, respectively (Dongiovanni et al. 2011). Under the iron overloading condition, the accelerated Fenton reaction and unquenchable consequence radicals occur by the availability of Fe^+2^ due to the elevated iron level of circulation resulting from hepcidin downregulation and also increased free iron (non-transferrin bind iron, NTBI) and loosely iron bonded to agents such as albumin, citrate and acetate in serum. The excessive ROS generation causes damage to the cellular macromolecules, lipids, protein, and nucleic acids, cell apoptosis followed by cytochrome c release resulting from mitochondrial membrane damage, and also ferroptosis, an iron-dependent regulated cell death. It has been demonstrated the pathological role of ROS and iron overload in the liver diseases (Capelletti et al. 2020). Indeed, iron overload and its accumulation in hepatocytes have recently been reported as common features of liver fibrosis, which could induce oxidative stress by proceeding of Fenton reaction, inflammation, and activation of HSCs. Also, iron deposition in Kupffer cells results in the production of pro-inflammatory cytokines, leading to liver fibrosis (Mehta et al. 2019). Iron overload occurs due to reducing or suppressing the maser systemic iron regulator, hepcidin. Iron plays a vital role in the viability of organisms due to its involvement in biological processes such as respiration, cell growth, and differentiation (Lu et al. 2016). Hence, a regulating iron level mechanism is mediated by hepcidin to control iron load via interaction with ferroportin (FPN), expressed by the leading iron stores such as enterocytes, macrophages, and hepatocytes (Atanasiu et al. 2007). This evidence implies that the altered gene expression profile of hepatocytes, especially hepcidin, can induce fibrogenic processes (such as activation of HSCs) beyond the inflammatory stimuli.

Hepcidin is a master iron regulator hepatokine derived mainly from hepatocytes, first identified by antimicrobial activity as Hepcidin Antimicrobial Peptide (HAMP) or Liver-expressed Antimicrobial Peptide-1 (LEAP-1). Hepcidin can inhibit *Escherichia coli, Salmonella typhimurium*, and *Mycobacterium tuberculosis* growth (Park et al. 2001, Sow et al. 2007, Nairz et al. 2008). It has been reported that a decrease in iron level in serum resulting from hepcidin induction, reduces the expression of outer membrane protein A (OmpA) of *Aeromonas hydrophila* in zebrafish model. OmpA which is induced by iron acts as a strategy to escape from the complement system in *A. hydrophila* invasion. These findings implicated the importance of iron to bacterial growth and expression of virulence factors such as OmpA. Therefore, the combined antibacterial and iron regulatory effects of hepcidin on bacterial defense against innate immunity can reduce bacterial invasion (Smith et al. 2007, Xiong et al. 2010, Michels et al. 2015, Jiang et al. 2017).

Furthermore, hepcidin levels are inversely related to iron load due to its ability to degrade ferroportin (FPN), encoded by *SLC40A1*, the principal iron efflux pump expressed on enterocytes, macrophages, and hepatocytes (Rice et al. 2009).

Hepcidin could regulate iron absorption mediated by enterocytes, primarily through regulating FPN stability on these cells. Enterocytes mediate iron absorption and circulation via several proteins, including duodenal cytochrome B (DCYTB) to reduce ferric iron to ferrous, divalent metal transporter 1 (DMT1) to transfer ferrous iron to enterocytes, FPN to export ferrous iron to circulation, hephaestin (HEPH) to oxidize ferrous iron to ferric, and transferrin (TF) as an iron plasma carrier (Fuqua et al. 2012). It is noticed that FPN is necessary for iron absorption. It has been showed that iron body load affects FPN expression so that it is inversely expressed in response to iron deficiency in enterocytes. Meanwhile, the expression of FPN in other tissues is differently regulated by the iron load. For example, liver-expressed FPN is decreased and increased by iron deficiency and iron overload, respectively. This is a protective regulatory mechanism against intracellular toxic iron accumulation (De Domenico et al. 2006, Bogdan et al. 2016, Link et al. 2021).

Macrophages and hepatocytes are also the targets of hepcidin to control iron efflux due to their roles in hemoglobin recycling and primary storage of iron, respectively. The intracellular overload of iron in hepatocytes results from the downregulation of hepcidin, which is followed by the incensement of iron efflux from macrophages and enterocytes to circulation due to lacking inhibitory function on FPN activity (Kessler et al. 2015, Mehta et al. 2019).

**Figure 1.**
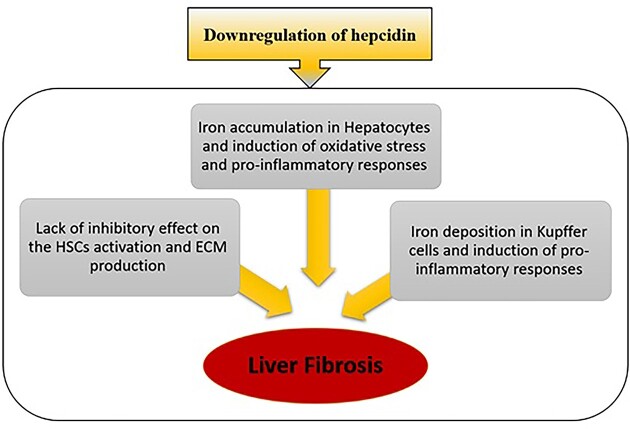
The effects of downregulated hepcidin in induction of liver fibrosis.

Furthermore, the role of hepcidin in the intercellular communication of liver cells, especially hepatocytes and HSCs, was identified in a fantastic study. Han Yeob Ch et al. showed the inhibitory role of hepcidin in the activation of HSCs by degradation of FPN (expressed on HSCs) and improvement of liver fibrosis. Indeed, FPN deficiency in activated HSCs suppresses the phosphorylation of SMAD3, induced by TGF-β signaling via hepcidin overexpression in CCl4-induced fibrotic mice. As a result, the suppression of HSC activity disrupts the production of ECM and inflammatory cytokines, elevating hepatocytes' injury and fibrosis (Han et al. 2016). On the other hand, disrupted iron homeostasis contributes to HSC activation. Unlike quiescent, activated HSCs express two iron-related receptors, including a specific receptor for the H-ferritin receptor and transferrin receptor-1 (TFR-1) (Mehta et al. 2019). H-ferritin, released from Kupffer cells after hemoglobin recycling from senescent RBC (erythrophagocytosis), binds to the H-ferritin receptor and internalizes to activated HSCs (Mao et al. 2015). Subsequently, proinflammatory and profibrogenic effects are exerted by activating nuclear factor-kappa B (NF-κB) and elevated IL-6 and IL-1β due to free radical production. Also, TFR-1 activation of activated HSCs with transferrin increases the transcripts of ECM components, such as α-smooth muscle actin (α-SMA) and procollagen α1 (Bridle et al. 2003, Mehta et al. 2018). Interestingly, HSCs produce excessive ECM and cross-linked mature collagens during liver fibrosis, which are more resistant to MMP degradation. It has been demonstrated that iron overload may enhance fibrogenesis due to acting as a cofactor for hydroxylase enzymes catalyzing crosslinking collagens (Risteli and Kivirikko 1974). Generally, we presented the main effects of downregulated of hepcidin in liver fibrosis (Fig. 1).

### Regulation of Hepcidin Expression

According to the pivotal role of hepcidin in managing iron homeostasis and pathological consequences, its gene expression is tightly controlled by several mechanisms, including iron load and inflammation. Iron deficiency and increased erythropoiesis can decrease hepcidin expression, which is followed by the elevation of iron absorption and efflux of iron from storing cells to circulation. In contrast, iron overload upregulates hepcidin expression, decreasing iron absorption and keeping excess iron in macrophages and hepatocytes (Ganz and Nemeth 2012). Hereditary hemochromatosis is a known iron loading disorder due to HAMP mutations, emphasizing hepcidin's crucial role in responding to iron status. The severe form of iron overload is called juvenile hemochromatosis, resulting from mutation in the hemojuvelin (HJV) gene. HJV mediates one of the main inducing hepcidin expression signaling by bone morphogenetic proteins (BMP)/SMAD pathway against iron load. HJV acts as a BMP coreceptor (Xia et al. 2008). BMP6 is predominantly released by Liver Sinusoidal Endothelial Cells (LSECs) and HSCs in response to iron overload for the regulation of iron hemostasis through HAMP induction in hepatocytes. After BMP6 binds to its receptor and coreceptor, a complex of phosphorylation of SMAD 1/5/8 incorporated with SMAD4 is formed and translocated to the nucleus to induce HAMP promoter (Parrow and Fleming 2014, Xiao et al. 2020). In addition, Hemochromatosis Protein (HFE) and Transferrin Receptor 2 (TFR2), which are expressed on the plasma membrane of hepatocytes, sense plasma iron levels. Iron overload induces HFE and TFR2 to upregulate HAMP by influencing the BMP/SMAD signaling for controlling the iron circulation. The hemochromatosis may result from HFE and TFR2 mutations in relatively mild phenotypes (Chen et al. 2016).

Another main regulatory hepcidin expression is mediated by JAK (Janus kinase)–STAT3 (signal transducer and activator of transcription 3) pathway activated via inflammatory stimuli for reducing serum iron levels. Since iron is a pivotal element for the survival and replication of microorganisms, its suppression during infection is a defense strategy to sequester iron from microbial agents (Wang and Babitt 2016). In the inflammatory state, macrophages, monocytes, and Dendritic Cells (DCs) preserve intracellular iron as a form binding to ferritin and inhibiting iron export meditated by hepcidin (Cairo et al. 2011). Therefore, hepcidin acts as innate immunity and is categorized as a type II acute-phase protein. Elevated hepcidin during infection causes anemia of inflammation (AI) to diminish the viability of infectious agents. Hence, patients with a high iron level have a poor disease prognosis and a higher mortality rate (Lan et al. 2018). The main inducer cytokine to hepcidin expression is IL-6, which interreacts with IL-6 receptor GP-130 and induces phosphorylation and nucleus translocation of STAT-3. This role of IL-6 depends on macrophages expressing toll-like receptors (TLRs) but not released from Kupffer cells since depletion of the liver from Kupffer cells does not affect hepcidin expression by IL-6 exposure (Lou et al. 2005). Interestingly, hepcidin expression reduction was reported in biliary atresia (cholestatic injury). Hydrophobic bile acid disrupted the IL-6-STAT3 pathway to induce HAMP in hepatocytes by inhibiting STAT-3 phosphorylation (Huang et al. 2009).

Furthermore, a macrophage-independent manner has been described to induce hepcidin expression in hepatocytes. In this way, the activation of TLR-4 expressed on hepatocytes (instead of macrophages) induces the HAMP promotor. Lipopolysaccharide (LPS) has a proinflammatory activity on macrophages expressing TLR-4 to induce NF-kB and its targeted proinflammatory genes, such as IL-6, which is the main inflammatory inducer of hepcidin expression as a macrophage-mediated pathway (Lee et al. 2017). Also, LPS could interact with hepatocyte-expressed TLR-4 to induce the myeloid differentiation factor 88 (MyD88) pathway by activating c-Jun N-terminal kinase (JNK) and activator protein-1 (AP-1). The binding site to AP-1 is identified on the HAMP promotor. Therefore, LPS induces HAMP via the TLR-4-JNK-AP-1 axis in hepatocytes. In contrast, the suppression of liver hepcidin expression was reported by alcohol through TLR-4 and activation of NF-kB even in the presence of inflammatory stimuli (Zmijewski 2014). Also, LPS can elevate SMAD signaling to induce HAMP promoters by activating SMAD4, followed by the TLR-4-MyD88 pathway (Kowdley et al. 2021). Different adaptor molecules and transcription factors located downstream of TLR-4 explain different signaling pathways by various stimuli.

### Hepcidin Role in Chronic Liver Disease and Hepatocellular Carcinoma

Chronic Liver Disease (CLD) includes alcoholic/non-alcoholic liver disease and viral hepatitis, resulting in the dysfunction of inflammatory response and liver structure and progressing into fibrosis, cirrhosis, and Hepatocellular Carcinoma (HCC). Hepatic iron overloading is a common feature of CLD (Milic et al. 2016). Alcoholic Liver Disease (ALD) is a threat to global public health. In ALD, ethanol abuse promotes hepatic iron overload by inhibiting hepcidin gene expression. This inhibition is caused by inhibiting enhancer-binding protein (C/EBP), which results from alcohol and iron-mediated oxidative stress (Ferrao et al. 2022, Li et al. 2022). Also, alcohol can upregulate enterocyte DMT-1 and FPN, increasing serum iron and promoting liver fibrosis (Harrison-Findik et al. 2007).

Non-alcoholic Fatty Liver Disease (NAFLD), characterized by increased liver fat accumulation, is the most common liver disease worldwide. It consists of intracellular fat accumulation and steatosis, from simple to progressive, called non-alcoholic steatohepatitis (NASH), possibly progressing into liver fibrosis, cirrhosis, HCC, and death. There are pathogenic factors to the onset and development of NAFLD, including metabolic syndrome-related features such as obesity, insulin resistance, hypertension, hyperlipidemia, and hepatic iron overload (Younossi et al. 2020, Pantano et al. 2021). Iron overload can promote hepatic fibrosis, which is a NASH character. According to the complexity of NASH pathogenies and the proven role of iron load in liver fibrosis, reducing iron load can be a strategy to reduce steatosis, inflammation, and fibrosis in NASH treatment. In this regard, Chen et al. demonstrated a potential therapeutic role for hepcidin in alleviating steatohepatitis and fibrosis in NASH-induced animal models via recombinant adeno-associated virus genome 2 serotype 8 vector expressing Hamp (rAAV2/8-Hamp)-mediated hepcidin intervention. They reported that the overexpression of HAMP significantly improved liver fibrosis by suppressing proinflammatory response, infiltration of macrophages, and HSCs activation in a mouse model of NASH induced by a choline-deficient l-amino acid-defined (CDAA) diet (Chen et al. 2022). Furthermore, several studies demonstrated a significant correlation between serum hepcidin level, hepatic iron, and fibrosis in NAFLD/NASH patients (Valenti et al. 2010, Nelson et al. 2011, Nelson et al. 2012). Lu et al. emphasized the role of iron and hepcidin in the severity of NAFLD. They reported the role of hepcidin in regulating metabolic processes and lipid and carbohydrate metabolism in high fat-fed and high sucrose-fed *Hamp1* knockout mice (Lu 2016).

Hepatitis B Virus (HBV) and Hepatitis C Virus (HCV) are causative infectious factors in liver fibrosis, demonstrating disrupted hepcidin levels. Interestingly, HCV is a stronger oxidative stress inducer than other viral hepatitis agents. Also, HCV-induced liver fibrosis inhibits hepcidin expression by binding impairing C/EBP and STAT3 hepcidin promoters. Furthermore, HCV-induced hepcidin suppression is due to the antiviral activity inhibiting HCV replication. Therefore, antiviral therapy, which increases STAT3 expression level, could effectively restore hepcidin levels and decrease viral loads (Barry et al. 2009, Vela 2018).

As mentioned above, hepatic iron overload could lead to fibrosis, cirrhosis, and HCC due to the hepatocarcinogenic potential of iron. Contrary to other types of cancer in which the hepcidin level elevates, hepcidin is downregulated in HCC (Fan et al. 2021, Joachim and Mehta 2022). Evidence shows that an iron supplementation diet correlates with neoplastic hepatic nodules and HCC in animal and clinical models (Bothwell et al. 1964, Asare et al. 2006). Although the increased level of hepcidin inducer is seen in HCC, various mechanisms can be considered for downregulated HAMP expression in HCC. They may include the hypermethylation of HAMP promoters and BMP6 as potent hepcidin inducers, which are down-regulated in HCC (He et al. 2014, Udali et al. 2018). Additionally, there may be downregulation of TFR2 and HJV as iron sensing mediators that induce hepcidin (Maegdefrau et al. 2011, Joachim and Mehta 2022). Iron overload can lead to the downregulation of the tumor suppressor P53, which in turn decreases HAMP expression due to the presence of P53 response on HAMP promoters. Another possible factor is a mutation on TP53, the gene encoding P53, which activates HAMP transcription (Hussain et al. 2007, Shen et al. 2014). Also, there may be an elevation of matriptase-2 expression, which acts as a negative regulator for hepcidin expression (Lofft et al. 2020).

The downregulation of hepcidin in HCC can affect HCC pathogenesis by promoting the growth of cancerous cells by activating the cyclin-dependent kinase-1/STAT3 (CDK1/STAT3) pathway (Shen et al. 2019). Liver fibrosis and cirrhosis are significant factors in HCC development. Therefore, the downregulation of hepcidin in HCC can eliminate the protective effect of hepcidin because it lacks the inhibitory effect on TGF-β-induced smad3 phosphorylation, which inhibits HSC activation (Joachim and Mehta 2022). Also, increased BMP expression and activated BMP-SMAD signaling can influence HCC metastasis and invasion by promoting cancer cell migration (Maegdefrau and Bosserhoff 2012). The potential of hepcidin as a diagnostic, prognostic, and therapeutic factor has been suggested (Joachim and Mehta 2022). There is a challenge concerning HCC diagnosis, which affects prognosis, treatment, and cost burden. HCC is mainly diagnosed by assessing serum Alpha-fetoprotein (AFP) levels and imaging techniques. Approximately half of HCC patients exhibit AFP-negative tests, emphasizing the lack of comprehensive HCC biomarkers (Wang and Zhang 2020). Joachim JH et al. highlighted the potential of hepcidin as a new diagnostic biomarker for HCC (Joachim and Mehta 2022). Nahon P et al. reported an association between low levels of hepcidin and higher risks for HCC and poor prognosis in alcoholic cirrhotic patients (Nahon et al. 2016). Furthermore, a study reported the beneficial role of deferasirox (DFX) iron chelating against HCC (Saeki et al. 2016). Therefore, the modulation of iron level and hepcidin could be a target for HCC treatment.

### Microbiota-Gut-liver Axis and Liver Fibrosis

Gut microbiota is a co-evolved complex microbial population that colonizes the gastrointestinal (GI) tract. Currently, the pivotal role of gut microbiota in determining health and disease state has well been known (Fan and Pedersen 2021). Many studies implicate the involvement of the dysbiotic gut microbiota in the pathology of a wide range of diseases, including the most common liver diseases such as chronic hepatitis B (CHB), chronic hepatitis C (CHC), alcoholic liver disease (ALD), non-alcoholic fatty liver disease (NAFLD), non-alcoholic steatohepatitis (NASH), liver cirrhosis, and hepatocellular carcinoma (HCC) (Lin et al. 2014, Wieland et al. 2015, Wang et al. 2018, Álvarez-Mercado et al. 2019). Besides the regulatory role of gut microbiota in the modulation of various mechanisms, such as immune and metabolism pathways in the GI tract, it bi-directionally communicates with extraintestinal organs, especially the liver named the gut-liver axis. According to the anatomical and functional similarities between the GI tract and liver, their bidirectional interaction has been established to be primarily mediated via portal circulation (Adolph et al. 2018). The role of gut microbiota in the maintenance of normal liver function could be summarized in several main mechanisms, including (i) the modulation of systemic inflammatory responses due to the presence of nearly 70% of immune cells in lamina propria that is educated by lumen sampling of microbiota-derived immunological components (Ahmadi Badi et al. 2021), (ii) regulation of permeability of intestinal epithelium layer that determines pathogenic and symbiotic bacteria and their immunological components' translocation to lamina propria and circulation (Régnier et al. 2021), (iii) the influence on the bile acids (BAs) composition and pool size through deconjugation of primary to secondary BAs and BAs-farnesoid X receptor (FXR) interaction modulating metabolic and immune pathways and BAs production (Sayin et al. 2013) and (iv) the control of HSCs transdifferentiation by modulating the intrahepatic immune microenvironment (composition of immune cells and cytokines/chemokines profile) (Liang et al. 2020).

The liver continuously is exposed to antigen-derived nutrients, pathogens, microbiota, and their metabolites (such as short-chain fatty acids (SCFAs)), which reach the liver by the portal vein. In normal conditions, LPS derived from Gram-negative members of gut microbiota is translocated to the liver (by portal vein) and detoxified and cleared through the phagocytic activity of Kupffer cells in the reticuloendothelial system (RES) (Leber et al. 2012). Under liver injury conditions, the mentioned mechanism is disrupted due to the dysfunction of the gut barrier and the reduction of RES activity, finally resulting in the dominancy of proinflammatory responses. On the other hand, increased gut microbiota-derived LPS promotes HSCs transdifferentiation by activating TLR-4 expressed on HSCs, followed by the incensement of cytokines and chemokines (Zheng and Wang 2021). In addition, in the normal state, SCFAs derived from gut microbiota help maintain the normal liver function through several routes including the reinforcement of gut barrier integrity and control of bacterial translocation to the liver, acting as a signaling molecule to interact with G-protein-coupled receptor (GPR) 41, GPR43 and peroxisome proliferator-activated receptor γ (PPAR-γ), which mediate immune and metabolic homeostasis, having the epigenetic potential to the improvement of regulatory T-cells (Treg) by inhibiting histone deacetylase (HDAc) (Koh et al. 2016, Zheng and Wang 2021). In liver injury, the optimal concentration of SCFAs is disrupted, followed by the dysbiosis of gut microbiota composition, especially Bacteroidetes and Firmicutes phyla, including Ruminococcaceae, Lachnospiraceae, and Clostridiales, which are the main SCFAs producers (Furusawa et al. 2013, Zhang et al. 2021). Therefore, the gut-liver axis is negatively influenced by a liver injury that induces dysbiosis of gut microbiota and vis versa, which, in turn dysbiosis of gut microbiota could cause liver damage.

### Interplay between Gut Microbiota and Iron Homeostasis from Intestinal Iron absorption to Hepatic Hepcidin Expression

Iron derived from diet and hem could influence gut microbiota composition and metabolic activity. It has been demonstrated that very low iron condition negatively affects gut microbiota health by decreasing butyrate-producing bacteria, such as *Roseburia* spp. and *Bacteroides* spp. because Fe acts as a cofactor for enzyme involvement in the fermentation pathway (Dostal et al. 2013). Furthermore, Firmicute abundance, the main gut microbiota phylum, has been reported to increase and decrease under iron supplementation and iron deficiency conditions, respectively (Dostal et al. 2012, Dostal et al. 2013). Also, hem enriched intestinal lumen resulting from a hem-rich diet or intestinal bleeding may induce dominancy of bacteria containing the heme-uptake coding genes (Constante et al. 2017). There is an ongoing competition for iron acquisition between microbes and hosts mainly mediated by the release of iron-chelating proteins to the intestinal lumen, including siderophore and lipocalin-2, respectively (Chieppa and Giannelli 2018). Therefore, iron availability may be a pivotal regulator in preserving the symbiotic relationship between gut microbiota and the host.

Evidence emphasizes the role of gut microbiota in liver pathophysiology concentrated on iron homeostasis mediated by hepcidin. Hence, it is necessary to consider the mechanisms influenced by gut microbiota to modulate iron metabolism and hepcidin regulation for liver fibrosis control. On the one hand, the gut microbiota could affect iron metabolism due to the absorption of dietary iron in the GI tract colonized by this microbial community. Although the duodenum is the primary site for iron absorption (nearly 15% absorption rate), the rest of the iron reaches the colon, which is the leading site for the gut microbiota population (Yilmaz and Li 2018). The availability and valency of iron could be influenced by microbiota composition via the production of siderophores (chelating molecules for the acquisition of iron for bacterial cells) and SCFAs that can increase iron absorption by several mechanisms, including the supply of energy for the proliferation of epithelial cells, reduction of iron to the ferrous form, pH dropping of GI tract, and increment of iron solubility (Salovaara et al. 2003, Yilmaz and Li 2018). There is a correlation between gut microbiota-derived vitamins including vitamin B12 (cobalamin) and folate with erythropoiesis since their deficiency can lead to anemia (Koury and Ponka 2004). Furthermore, it has been reported that Vitamin B such as cobalamin affects gut microbiota composition and its capacity to produce SCFAs (Wan et al. 2022). Therefore, gut microbiota-derived vitamins such as vitamin B12 can influence the crosstalk between iron homeostasis and gut microbiota by the mentioned effects. The iron storage and expression of intestinal iron absorption/exporter-mediated proteins, such as DMT1, DCYTB, and FPN, could be controlled by gut microbiota. A study demonstrated that germ-free (GF) mice had significantly higher and lower levels of absorptive iron proteins (DMT1 and DCYTB) and iron efflux FPN than those colonized with microbiota. Also, *Bacteroides thetaiotaomicron, Faecalibacterium prausnitzii*, and probiotic strains (*Streptococcus thermophilus* LMD-9) could induce ferritin storage in the colon (Deschemin et al. 2016). Furthermore, it has been reported that *Lactobacillus* species are the main gut microbiota to sense iron levels and reduce host iron absorption. Gut microbiota metabolites, such as 1,3-diaminopropane (DAP) and reuterin, regulate iron homeostasis and ameliorate tissue iron overload by acting as a suppressor of hypoxia-inducible factor 2a (HIF-2a), a transcription factor of three key intestinal iron transporters (DMT1, DCYTB, and FPN), and increament of iron storage ferritin (Das et al. 2020). Interestingly, intestinal HIF-2a activation has been shown that is in association with the hepcidin/FPN axis to control iron absorption (Schwartz et al. 2019).

On the other hand, a study reported the inducing effect of gut microbiota on hepatocyte hepcidin expression. It identified the ability of *Bifidobacterium longum* and *Bacteroides fragilis* to upregulate hepcidin in a macrophage-stimulated manner which is mediated by activation of BMP/SMAD signaling with IL-1β. Also, the novel association between IL-1β and BMP/SMAD signaling to induce hepcidin and possibly different hepcidin induction mechanisms between humans and mice (the role of IL-1β) were suggested (Shanmugam et al. 2015).

Also, a fantastic study reported the effect of gut microbiota on the induction of hepcidin produced by non-hepatocyte sources. Bessman NJ reported a protective effect of gut microbiota on mucosal healing through the induction of hepcidin production from conventional dendritic cells (cDCs), which are essential to repair tissue by hepcidin production in the inflamed intestine. The cDCs produce hepcidin in response to gut microbiota stimuli. This limits the availability of local intestinal iron by sequestering it by targeting intestinal phagocytes that express FPN. It also helps to limit tissue infiltration. This hepcidin, produced from cDC, has been characterized and compared with hepcidin derived from the liver, which is induced by inflammatory responses and provides protection against systemic infection (Bessman et al. 2020).

The bidirectional crosstalk between gut microbiota members and the host for iron acquisition and metabolism plays an important role in shaping the metabolism of both the host and the gut microbiota. As discussed above, the potential crosstalk is mediated by the influence of bacterial species on iron metabolism and the impact of nutrient-derived iron on gut microbiota composition. This interaction affects iron levels and storage, the immune system, and glucose metabolism, which is related to metabolic syndrome (Mayneris-Perxachs et al. 2022). Therefore, there is a correlation between iron and glucose metabolism due to disrupted iron homeostasis and changes in gut microbiota composition in patients with metabolic disease and insulin resistance (Fillebeen et al. 2020). Several studies have reported that iron overload is a risk factor for diabetes (Simcox and Mcclain 2013, Fernández-Real and Manco 2014). There is an interconnection between iron and glucose metabolism, which are hormonally regulated by hepcidin and insulin, respectively. In this regard, documents represent the direct induction effect of hepcidin via STAT3 on hepatocytes. Additionally, there is evidence of increased hepcidin levels due to glucose intake in healthy subjects (Aigner et al. 2013, Wang et al. 2014). Accordingly, metabolic syndrome, such as type 2 diabetes, is associated with altered gut microbiota and iron metabolism. A study supports that the improvement of glucose levels may be controlled by Salidroside (SAL), a Chinese herbal compound with the potential to affect gut microbiota composition and protect against iron overloading. This is achieved by targeting gut microbiota and iron metabolism in diabetic mice (Shi et al. 2022). Therefore, considering the putative correlation could be useful for designing therapeutic strategies for metabolic syndrome.

**Figure 2.**
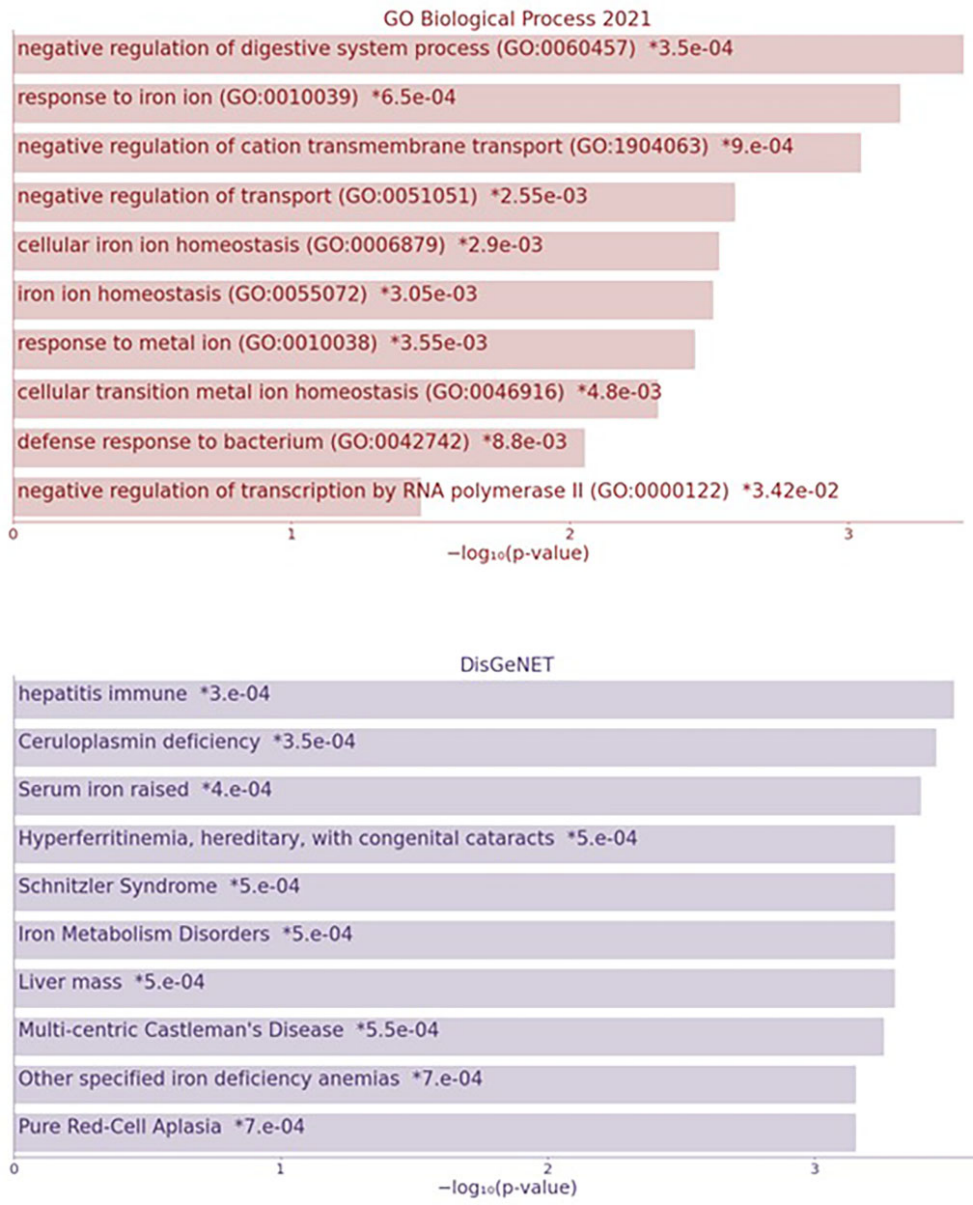
The bar plot of the HAMP gene in A) biological processes and B) pathogenic phenotypes. In biological processes, iron metabolism, positive and negative self-regulation in the formation of hemoglobin by this gene are highly evident. Also, in diseases related to liver fibrosis, there has been a more significant observation of liver mass and iron metabolism in the liver.

Using primary and integrated bioinformatics analysis, we investigated the HAMP gene and identified its role in biological processes and the types of diseases caused by defects in this gene. Next, we represented the bacteria interacting with the HAMP gene as a scatter diagram. These findings revealed that this gene plays a significant role in iron-dependent metabolic processes and is associated with diseases such as thalassemia, liver fibrosis, immune system disorders in the liver, iron metabolism disorders, and aplasia in red blood cells (Fig. 2).

**Figure 3.**
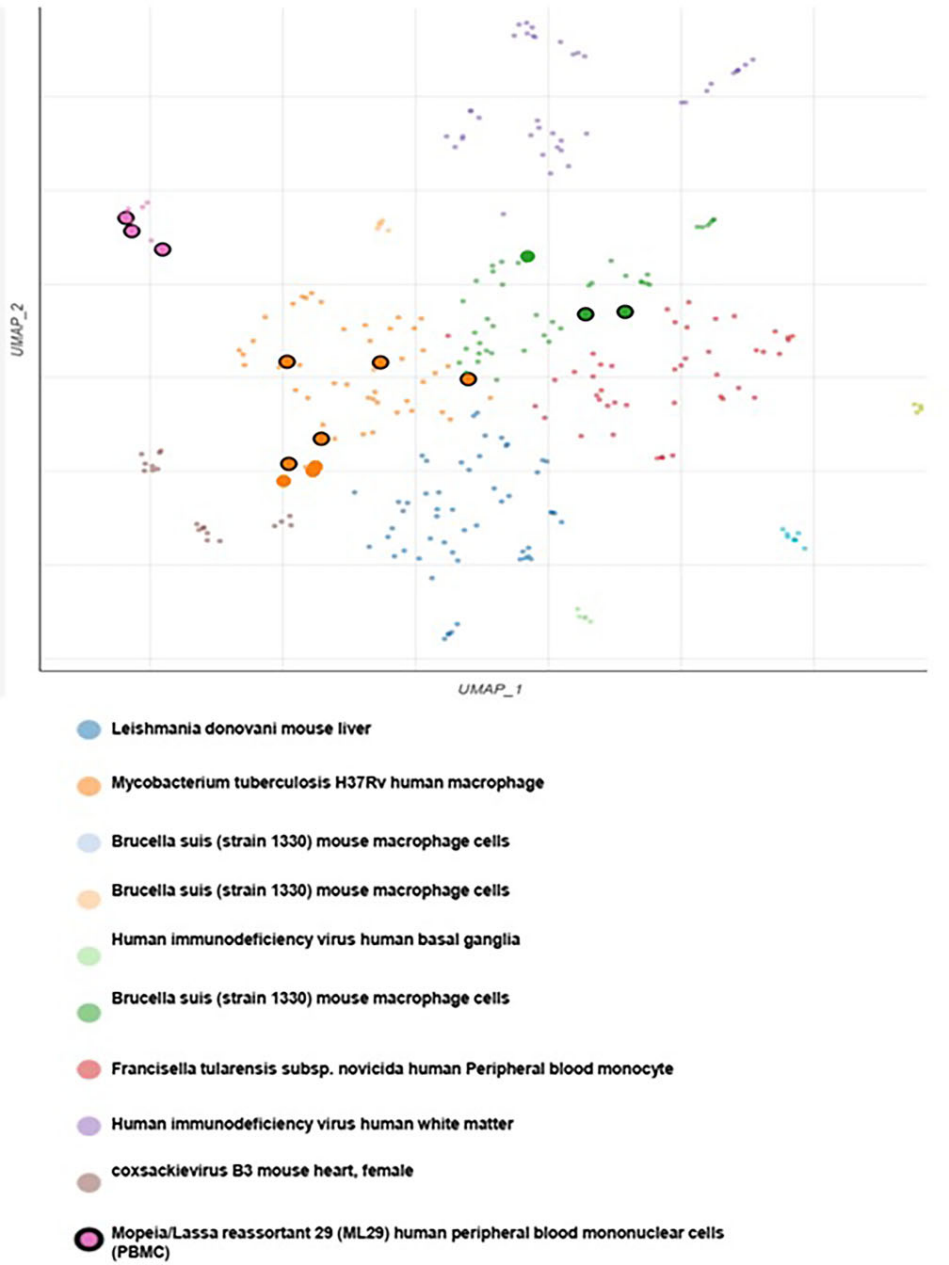
The scatter plot of the degree of accumulation and the correlation of HAMP expression with various bacterial strains and microbes.

In the next part of the analysis, we found that the HAMP gene is significantly associated with bacterial strains such as Mycobacterium tuberculosis and *Brucella suis*. Additionally, HAMP is related to other microorganisms such as Coxsackievirus, Human Immunodeficiency Virus, and Leishmania (Fig. 3).

**Figure 4.**
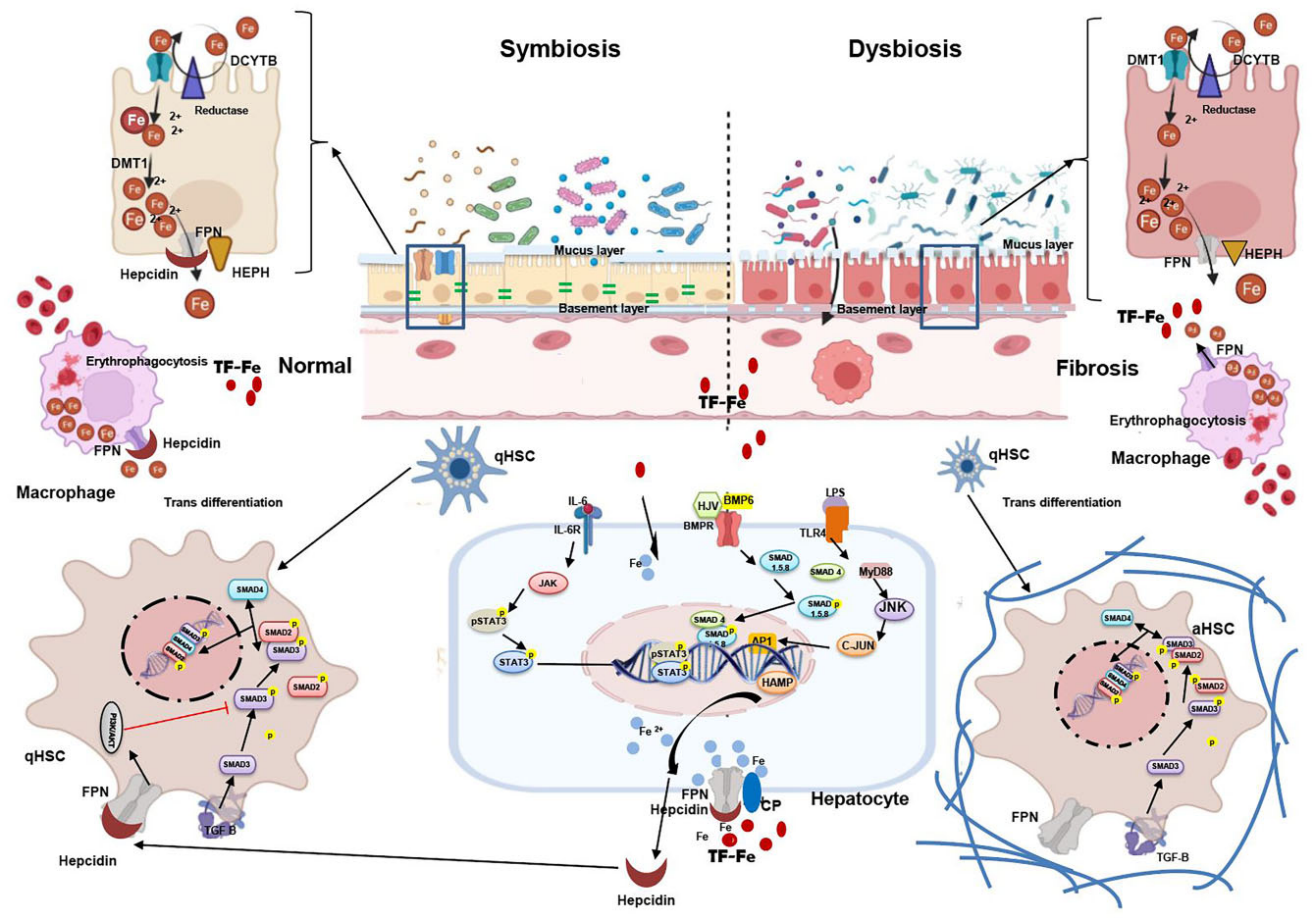
The possible interaction between gut microbiota and hepatic hepcidin expression in the determination of normal liver function and pathological fibrosis; in a symbiosis state, the liver has normal functioning due to fine-tuning of hepcidin gene expression in hepatocytes. Hepcidin controls liver physiology by suppressing HSCs activation (by degrading FPN and suppressing SMADs signaling to produce ECM) and inhibiting intracellular hepatic iron overload through inhibiting iron over-efflux from enterocytes and macrophages to circulation. Also, intestinal iron absorption could be desired by the normal function of DMT-1 and DCYTB and an intact gut barrier to inhibit the over-translocation of immunological components to lamina propria and circulation, which control inflammatory signals in the liver. Gut microbiota components, such as LPS, could regulate hepcidin expression by direct (stimulation of TLR-4) and macrophage-mediated (activation of IL-6 receptor) effects in hepatocytes. Under a dysbiotic state, the perturbed gut barrier function increases bacterial translocation, inducing pro-inflammatory responses, and exacerbating hepatic fibrogenic reactions. The inhibitory hepcidin effect on the activated HSCs is disrupted which results to elevated activation of HSCs and excessive accumulation of ECM. On the other hand, the plasma iron level and hepatic intracellular iron overload could be affected by gut microbiota, their components, and metabolites via influencing the expression of iron abortive proteins, such as DMT-1 and DCYTB, and iron efflux pump, FPN.

According to the crucial role of gut microbiota in driving the gut-liver axis, bacterial translocation to the liver and possible influential direct and macrophage-mediated effects on hepcidin expression could be rationally based on the stimulation of systemic immunity and continued exposure of the liver to gut microbiota components and metabolites. Taken together, we summarized the possible crosstalk between gut microbiota and hepcidin expression to determine the physiology and pathophysiology of liver fibrosis (Fig. 4).

### Conclusion

We can illustrate the potential pathways for their interactions based on the role of gut microbiota in liver pathophysiology and iron metabolism, as well as the complexity of iron homeostasis and its prominent role in liver fibrosis. Gut microbiota may modulate hepatocyte hepcidin gene expression, especially via direct and macrophage-mediated effects. Furthermore, the intestinal expression of proteins mediating iron absorption and exportation may be regulated by gut microbiota composition. These data suggest the potential of the gut microbiota as hepatoprotective bacteria in controlling and modulating liver fibrosis by regulating hepcidin, which controls iron homeostasis. However, it is necessary to elucidate the molecular signaling pathways related to the fine-tuning control of iron levels and preserve the symbiotic relationship between the host and gut microbiota in liver fibrosis.

### Expert Opinion

Pathological liver fibrosis occurs due to chronic liver injury. Hepatotoxic and cholestatic liver injuries resulting from chronic viral infections, metabolic syndrome, and obstructive bile flow, such as biliary atresia, can progress to fibrosis, cirrhosis, and Hepatocellular Carcinoma (HCC). One of the main features of liver fibrosis is iron accumulation in hepatic cells, including hepatocytes. This accumulation can induce an oxidative stress response, inflammation, and activation of Hepatic Stellate Cells (HSCs), ultimately leading to the onset and development of fibrogenic responses. There is a proven role for hepcidin as the main regulator of iron in the pathophysiology of liver fibrosis by targeting FPN. The FPN activity involves the efflux of iron from the main iron donor and storage cells, including enterocytes, macrophages, and hepatocytes, into circulation. Hepatocyte iron accumulation is considered the starting point for the induction of inflammatory and fibrogenic responses resulting from decreased hepcidin levels.

In addition, fine-tuning intercellular communication between hepatic cells, such as hepatocytes, Kupffer cells, and HSCs, preserves the liver's normal function, which could be disrupted by hepcidin dysregulation. In contrast, it has been demonstrated that gut microbiota plays a pivotal role in regulating liver function through the gut-liver axis. Also, iron levels could alter gut microbiota composition and metabolic activity, affecting liver function. Some studies report the alteration of gut microbiota composition in liver injuries, such as liver fibrosis, which could be followed by cirrhosis and hepatocellular carcinoma. Therefore, the gut-liver axis is negatively influenced by liver injury, which can induce dysbiosis of the gut microbiota. Conversely, dysbiosis of the gut microbiota can also cause liver damage. Furthermore, the iron level, which is regulated by the hepcidin-FPN axis and proteins involved in iron uptakes, such as DCYTB and DMT1, could be influenced by the composition of gut microbiota, which can be altered by changes in iron levels.

Hepcidin expression is controlled by various factors, especially iron levels and the inflammatory response, which are correlated with maintaining the symbiotic relationship between the host and gut microbiota. Therefore, we can consider a powerful interplay between hepcidin and gut microbiota as two key factors in liver fibrosis. This delicate perspective could be considered when designing a gut microbiota-targeted intervention for regulating iron levels influenced by hepcidin activity. By considering the potential role of gut microbiota in modulating inflammation and hepatoprotective activities, it is necessary to understand the interplay between gut microbiota and hepcidin during liver fibrosis. Furthermore, we should consider that antifibrotic drugs may have side effects on normal hepatic cells. On the other hand, restoration of intercellular crosstalk between hepatic cells through modulation of hepcidin levels may offer a promising therapeutic strategy for preventing, controlling, and treating liver fibrosis. It has been documented that gut microbiota is pivotal in determining health and disease status, such as liver complications. Therefore, we should consider gut microbiota members as important actors in liver fibrosis therapeutic strategies to restore dysbiotic gut microbiota-host interactions. Targeted interventions for the gut microbiota and the discovery of novel components derived from the gut microbiota have shown promising potential in this field.

## Data Availability

The data used to support the findings in this study are available from the corresponding author upon reasonable request.

## References

[bib1] Adolph TE, Grander C, Moschen AR et al. Liver–microbiome axis in health and disease. Trends Immunol. 2018;39:712–23. 29843959 10.1016/j.it.2018.05.002

[bib2] Ahmadi Badi S, Tarashi S, Fateh A et al. From the role of microbiota in gut-lung axis to SARS-CoV-2 pathogenesis. Mediators Inflamm. 2021;2021:1–12. 10.1155/2021/6611222 PMC8059477 33953641

[bib3] Aigner E, Felder TK, Oberkofler H et al. Glucose acts as a regulator of serum iron by increasing serum hepcidin concentrations. J Nutr Biochem. 2013;24:112–7. 22819549 10.1016/j.jnutbio.2012.02.017

[bib4] Álvarez-Mercado A, Navarro-Oliveros M, Robles-Sánchez C et al. Microbial population changes and their relationship with human health and disease. Microorganisms. 2019;7:68. 30832423 10.3390/microorganisms7030068 PMC6463060

[bib5] Asare GA, Paterson AC, Kew MC et al. Iron-free neoplastic nodules and hepatocellular carcinoma without cirrhosis in Wistar rats fed a diet high in iron. J Pathol: J Patholog Soc Great Britain Ireland. 2006;208:82–90. 10.1002/path.1875 16278820

[bib6] Atanasiu V, Manolescu B, Stoian I. Hepcidin–central regulator of iron metabolism. Eur J Haematol. 2007;78:1–10. 17042775 10.1111/j.1600-0609.2006.00772.x

[bib7] Aydin MM, Akcali KC. Liver fibrosis. Turkish J Gastroenterol. 2018;29:14. 10.5152/tjg.2018.17330 PMC6322608 29391303

[bib8] Barry SP, Townsend PA, Mccormick J et al. STAT3 deletion sensitizes cells to oxidative stress. Biochem Biophys Res Commun. 2009;385:324–9. 19450559 10.1016/j.bbrc.2009.05.051 PMC2706948

[bib9] Bataller R, Brenner DA. Liver fibrosis. J Clin Invest. 2005;115:209–18. 15690074 10.1172/JCI24282 PMC546435

[bib10] Bessman NJ, Mathieu JRR, Renassia C et al. Dendritic cell–derived hepcidin sequesters iron from the microbiota to promote mucosal healing. Science. 2020;368:186–9. 32273468 10.1126/science.aau6481 PMC7724573

[bib11] Bogdan AR, Miyazawa M, Hashimoto K et al. Regulators of iron homeostasis: new players in metabolism, cell death, and disease. Trends Biochem Sci. 2016;41:274–86. 26725301 10.1016/j.tibs.2015.11.012 PMC4783254

[bib12] Bothwell TH, Seftel H, Jacobs P et al. Iron overload in Bantu subjects: studies on the availability of iron in Bantu beer. Am J Clin Nutr. 1964;14:47–51. 14106870 10.1093/ajcn/14.1.47

[bib13] Bridle KR, Crawford DHG, Ramm GA. Identification and characterization of the hepatic stellate cell transferrin receptor. Am J Pathol. 2003;162:1661–7. 12707050 10.1016/S0002-9440(10)64300-3 PMC1851195

[bib14] Cairo G, Recalcati S, Mantovani A et al. Iron trafficking and metabolism in macrophages: contribution to the polarized phenotype. Trends Immunol. 2011;32:241–7. 21514223 10.1016/j.it.2011.03.007

[bib15] Capelletti MM, Manceau H, Puy H et al. Ferroptosis in liver diseases: an overview. Int J Mol Sci. 2020;21:4908. 32664576 10.3390/ijms21144908 PMC7404091

[bib16] Chen H, Zhao W, Yan X et al. Overexpression of hepcidin alleviates steatohepatitis and fibrosis in a diet-induced nonalcoholic steatohepatitis. J Clin Transl Hepatol. 2022;10:577–88. 36062292 10.14218/JCTH.2021.00289 PMC9396326

[bib17] Chen S, Feng T, Vujić Spasić M et al. Transforming growth factor β1 (TGF-β1) activates hepcidin mRNA expression in hepatocytes. J Biol Chem. 2016;291:13160–74. 27129231 10.1074/jbc.M115.691543 PMC4933231

[bib18] Chieppa M, Giannelli G. Immune cells and microbiota response to iron starvation. Front Med. 2018;5:109. 10.3389/fmed.2018.00109 PMC5915481 29721497

[bib19] Constante M, Fragoso G, Lupien-Meilleur J et al. Iron supplements modulate colon microbiota composition and potentiate the protective effects of probiotics in dextran sodium sulfate-induced colitis. Inflamm Bowel Dis. 2017;23:753–66. 28368910 10.1097/MIB.0000000000001089

[bib20] Das NK, Schwartz AJ, Barthel G et al. Microbial metabolite signaling is required for systemic iron homeostasis. Cell Metab. 2020;31:115–30. e6. 31708445 10.1016/j.cmet.2019.10.005 PMC6949377

[bib21] De Domenico I, McVey Ward D, Nemeth E et al. Molecular and clinical correlates in iron overload associated with mutations in ferroportin. Haematologica. 2006;91:1092. 16885049 PMC3718284

[bib22] Deschemin J-C, Noordine M-L, Remot A et al. The microbiota shifts the iron sensing of intestinal cells. FASEB J. 2016;30:252–61. 26370847 10.1096/fj.15-276840

[bib23] Dongiovanni P, Fracanzani AL, Fargion S et al. Iron in fatty liver and in the metabolic syndrome: a promising therapeutic target. J Hepatol. 2011;55:920–32. 21718726 10.1016/j.jhep.2011.05.008

[bib24] Dostal A, Chassard C, Hilty FM et al. Iron depletion and repletion with ferrous sulfate or electrolytic iron modifies the composition and metabolic activity of the gut microbiota in rats. J Nutr. 2012;142:271–7. 22190022 10.3945/jn.111.148643 PMC3260059

[bib25] Dostal A, Fehlbaum S, Chassard C et al. Low iron availability in continuous in vitro colonic fermentations induces strong dysbiosis of the child gut microbial consortium and a decrease in main metabolites. FEMS Microbiol Ecol. 2013;83:161–75. 22845175 10.1111/j.1574-6941.2012.01461.x PMC3511601

[bib26] Fallowfield JA, Mizuno M, Kendall TJ et al. Scar-associated macrophages are a major source of hepatic matrix metalloproteinase-13 and facilitate the resolution of murine hepatic fibrosis. J Immunol. 2007;178:5288–95. 17404313 10.4049/jimmunol.178.8.5288

[bib27] Fan Y, Liu B, Chen F et al. Hepcidin upregulation in lung cancer: a potential therapeutic target associated with immune infiltration. Front Immunol. 2021;12:612144. 33868231 10.3389/fimmu.2021.612144 PMC8047218

[bib28] Fan Y, Pedersen O. Gut microbiota in human metabolic health and disease. Nat Rev Microbiol. 2021;19:55–71. 32887946 10.1038/s41579-020-0433-9

[bib29] Fernández-Real JM, Manco M. Effects of iron overload on chronic metabolic diseases. Lancet Diabetes Endocrinol. 2014;2:513–26. 24731656 10.1016/S2213-8587(13)70174-8

[bib30] Ferrao K, Ali N, Mehta KJ, Iron and iron-related proteins in alcohol consumers: cellular and clinical aspects. J Mol Med. 2022;100:1673–89. 36214835 10.1007/s00109-022-02254-8 PMC9691479

[bib31] Fillebeen C, Lam NH, Chow S et al. Regulatory connections between iron and glucose metabolism. Int J Mol Sci. 2020;21:7773. 33096618 10.3390/ijms21207773 PMC7589414

[bib32] Fuqua BK, Vulpe CD, Anderson GJ. Intestinal iron absorption. J Trace Elem Med Biol. 2012;26:115–9. 22575541 10.1016/j.jtemb.2012.03.015

[bib33] Furusawa Y, Obata Y, Fukuda S et al. Commensal microbe-derived butyrate induces the differentiation of colonic regulatory T cells. Nature. 2013;504:446–50. 24226770 10.1038/nature12721

[bib34] Ganz T, Nemeth E. Hepcidin and iron homeostasis. Biochim Biophys Acta. 2012;1823:1434–43. 22306005 10.1016/j.bbamcr.2012.01.014 PMC4048856

[bib35] Han CY, Koo JH, Kim SH et al. Hepcidin inhibits Smad3 phosphorylation in hepatic stellate cells by impeding ferroportin-mediated regulation of Akt. Nat Commun. 2016;7:1–14. 10.1038/ncomms13817 PMC5192182 28004654

[bib36] Harrison-Findik DD, Klein E, Crist C et al. Iron-mediated regulation of liver hepcidin expression in rats and mice is abolished by alcohol. Hepatology. 2007;46:1979–85. 17763462 10.1002/hep.21895

[bib37] He Y, Cui Y, Xu B et al. Hypermethylation leads to bone morphogenetic protein 6 downregulation in hepatocellular carcinoma. PLoS One. 2014;9:e87994. 24498236 10.1371/journal.pone.0087994 PMC3907571

[bib38] Huang Y-H, Chuang J-H, Yang Y-L et al. Cholestasis downregulate hepcidin expression through inhibiting IL-6-induced phosphorylation of signal transducer and activator of transcription 3 signaling. Lab Invest. 2009;89:1128–39. 19652645 10.1038/labinvest.2009.82

[bib39] Hussain SP, Schwank J, Staib F et al. TP53 mutations and hepatocellular carcinoma: insights into the etiology and pathogenesis of liver cancer. Oncogene. 2007;26:2166–76. 17401425 10.1038/sj.onc.1210279

[bib40] Jiang X-F, Liu Z-F, Lin A-F et al. Coordination of bactericidal and iron regulatory functions of hepcidin in innate antimicrobial immunity in a zebrafish model. Sci Rep. 2017;7:4265. 28655927 10.1038/s41598-017-04069-x PMC5487360

[bib41] Joachim JH, Mehta KJ. Hepcidin in hepatocellular carcinoma. Br J Cancer. 2022;127:185–92. 35264787 10.1038/s41416-022-01753-2 PMC9296449

[bib42] Kessler SM, Laggai S, Kiemer AK et al. Hepatic hepcidin expression is decreased in cirrhosis and HCC. J Hepatol. 2015;62:977–9. 25463544 10.1016/j.jhep.2014.10.046

[bib43] Khedr NF, Khedr EG. Branched chain amino acids supplementation modulates TGF-b1/smad signaling pathway and interleukins in CCl4-induced liver fibrosis. 10.1111/fcp.12297 28544244

[bib44] Kisseleva T, Brenner D. Molecular and cellular mechanisms of liver fibrosis and its regression. Nat Rev Gastroenterol Hepatol. 2021;18:151–66. 33128017 10.1038/s41575-020-00372-7

[bib45] Koh A, De Vadder F, Kovatcheva-Datchary P et al. From dietary fiber to host physiology: short-chain fatty acids as key bacterial metabolites. Cell. 2016;165:1332–45. 27259147 10.1016/j.cell.2016.05.041

[bib46] Koury MJ, Ponka P. New insights into erythropoiesis: the roles of folate, vitamin B12, and iron. Annu Rev Nutr. 2004;24:105–31. 15189115 10.1146/annurev.nutr.24.012003.132306

[bib47] Kowdley KV, Gochanour EM, Sundaram V et al. Hepcidin signaling in health and disease: ironing out the details. Hepatology Communications. 2021;5:723–35. 34027264 10.1002/hep4.1717 PMC8122377

[bib48] Lan P, Pan K-H, Wang S-J et al. High serum iron level is associated with increased mortality in patients with sepsis. Sci Rep. 2018;8:1–7. 30038422 10.1038/s41598-018-29353-2 PMC6056487

[bib49] Leber B, Spindelboeck W, Stadlbauer V. Infectious complications of acute and chronic liver disease. In Seminars in respiratory and critical care medicine. Thieme Medical Publishers. 2012;33:80–95. 10.1055/s-0032-1301737 22447263

[bib50] Lee Y-S, Kim YH, Jung YS et al. Hepatocyte toll-like receptor 4 mediates lipopolysaccharide-induced hepcidin expression. Exp Mol Med. 2017;49:e408. 29217822 10.1038/emm.2017.207 PMC5750473

[bib51] Li L-X, Guo F-F, Liu H et al. Iron overload in alcoholic liver disease: underlying mechanisms, detrimental effects, and potential therapeutic targets. Cell Mol Life Sci. 2022;79:201. 35325321 10.1007/s00018-022-04239-9 PMC11071846

[bib52] Liang Q, Zhang M, Hu Y et al. Gut microbiome contributes to liver fibrosis impact on T cell receptor immune repertoire. Front Microbiol. 2020;11: 3020. 10.3389/fmicb.2020.571847 PMC7729130 33329430

[bib53] Lin C-S, Chang CJ, Lu CC et al. Impact of the gut microbiota, prebiotics, and probiotics on human health and disease. Biomed J. 2014;37:259–68. 25179725 10.4103/2319-4170.138314

[bib54] Link C, Knopf JD, Marques O et al. The role of cellular iron deficiency in controlling iron export. Biochimica Et Biophysica Acta (BBA)-General Subjects. 2021;1865:129829. 33340587 10.1016/j.bbagen.2020.129829

[bib55] Lofft Z, Taibi A, Comelli E. Cranberry-derived proanthocyanidin and its gut microbial metabolites affect the intestinal miRNome in a distinct manner In vitro. Curr Develop Nutr. 2020;4:430.

[bib56] Lou D-Q, Lesbordes J-C, Nicolas G et al. Iron-and inflammation-induced hepcidin gene expression in mice is not mediated by Kupffer cells in vivo. Hepatology. 2005;41:1056–64. 15793843 10.1002/hep.20663

[bib57] Lu M, Li J, Zhang T et al. Serum biomarkers indicate long-term reduction in liver fibrosis in patients with sustained virological response to treatment for HCV infection. Clin Gastroenterol Hepatol. 2016;14:1044–55. e3. 26804385 10.1016/j.cgh.2016.01.009 PMC5726250

[bib58] Lu S . Lack of hepcidin expression attenuates steatosis and causes fibrosis in the liver. World J Hepatol. 2016;8:211. 26855692 10.4254/wjh.v8.i4.211 PMC4733464

[bib59] Maegdefrau U, Arndt S, Kivorski G et al. Downregulation of hemojuvelin prevents inhibitory effects of bone morphogenetic proteins on iron metabolism in hepatocellular carcinoma. Lab Invest. 2011;91:1615–23. 21863061 10.1038/labinvest.2011.123

[bib60] Maegdefrau U, Bosserhoff A-K. BMP activated Smad signaling strongly promotes migration and invasion of hepatocellular carcinoma cells. Exp Mol Pathol. 2012;92:74–81. 22024355 10.1016/j.yexmp.2011.10.004

[bib61] Mao Q, Xie Z, Wang X et al. Clonorchis sinensis ferritin heavy chain triggers free radicals and mediates inflammation signaling in human hepatic stellate cells. Parasitol Res. 2015;114:659–70. 25413629 10.1007/s00436-014-4230-0

[bib62] Mayneris-Perxachs J, Moreno-Navarrete JM, Fernández-Real JM. The role of iron in host–microbiota crosstalk and its effects on systemic glucose metabolism. Nat Rev Endocrinol. 2022;18:683–98. 35986176 10.1038/s41574-022-00721-3

[bib63] Mehta KJ, Coombes JD, Briones-Orta M et al. Iron enhances hepatic fibrogenesis and activates transforming growth factor-β signaling in murine hepatic stellate cells. Am J Med Sci. 2018;355:183–90. 29406047 10.1016/j.amjms.2017.08.012

[bib64] Mehta KJ, Farnaud SJ, Sharp PA. Iron and liver fibrosis: mechanistic and clinical aspects. World J Gastroenterol. 2019;25:521. 30774269 10.3748/wjg.v25.i5.521 PMC6371002

[bib65] Michels K, Nemeth E, Ganz T et al. Hepcidin and host defense against infectious diseases. PLoS Pathog. 2015;11:e1004998. 26291319 10.1371/journal.ppat.1004998 PMC4546197

[bib66] Milic S, Mikolasevic I, Orlic L et al. The role of iron and iron overload in chronic liver disease. Med Sci Monit. 2016;22:2144. 27332079 10.12659/MSM.896494 PMC4922827

[bib67] Nahon P, Nuraldeen R, Rufat P et al. In alcoholic cirrhosis, low-serum hepcidin levels associate with poor long-term survival. Liver Int. 2016;36:185–8. 26561367 10.1111/liv.13007

[bib68] Nairz M, Fritsche G, Brunner P et al. Interferon-γ limits the availability of iron for intramacrophage Salmonella typhimurium. Eur J Immunol. 2008;38:1923–36. 18581323 10.1002/eji.200738056

[bib69] Nelson JE, Brunt EM, Kowdley KV. Lower serum hepcidin and greater parenchymal iron in nonalcoholic fatty liver disease patients with C282Y HFE mutations. Hepatology. 2012;56:1730–40. 22611049 10.1002/hep.25856 PMC3462887

[bib70] Nelson JE, Wilson L, Brunt EM et al. Relationship between the pattern of hepatic iron deposition and histological severity in nonalcoholic fatty liver disease. Hepatology. 2011;53:448–57. 21274866 10.1002/hep.24038 PMC3058264

[bib71] Pantano L, Agyapong G, Shen Y et al. Molecular characterization and cell type composition deconvolution of fibrosis in NAFLD. Sci Rep. 2021;11:1–14. 34508113 10.1038/s41598-021-96966-5 PMC8433177

[bib72] Park CH, Valore EV, Waring AJ et al. Hepcidin, a urinary antimicrobial peptide synthesized in the liver. J Biol Chem. 2001;276:7806–10. 11113131 10.1074/jbc.M008922200

[bib73] Parrow NL, Fleming RE. Bone morphogenetic proteins as regulators of iron metabolism. Annu Rev Nutr. 2014;34:77–94. 24995692 10.1146/annurev-nutr-071813-105646 PMC7713507

[bib74] Ramachandran P, Pellicoro A, Vernon MA et al. Differential Ly-6C expression identifies the recruited macrophage phenotype, which orchestrates the regression of murine liver fibrosis. Proc Natl Acad Sci. 2012;109:E3186–95. 23100531 10.1073/pnas.1119964109 PMC3503234

[bib75] Régnier M, Van Hul M, Knauf C et al. Gut microbiome, endocrine control of gut barrier function and metabolic diseases. J Endocrinol. 2021;248:R67–82. 33295880 10.1530/JOE-20-0473

[bib76] Rice AE, Mendez MJ, Hokanson CA et al. Investigation of the biophysical and cell biological properties of ferroportin, a multipass integral membrane protein iron exporter. J Mol Biol. 2009;386:717–32. 19150361 10.1016/j.jmb.2008.12.063 PMC2677177

[bib77] Risteli J, Kivirikko KI. Activities of prolyl hydroxylase, lysyl hydroxylase, collagen galactosyltransferase and collagen glucosyltransferase in the liver of rats with hepatic injury. Biochem J. 1974;144:115–22. 4376954 10.1042/bj1440115 PMC1168471

[bib78] Saeki I, Yamamoto N, Yamasaki T et al. Effects of an oral iron chelator, deferasirox, on advanced hepatocellular carcinoma. World J Gastroenterol. 2016;22:8967. 27833388 10.3748/wjg.v22.i40.8967 PMC5083802

[bib79] Salovaara S, Sandberg A-S, Andlid T. Combined impact of pH and organic acids on iron uptake by Caco-2 cells. J Agric Food Chem. 2003;51:7820–4. 14664552 10.1021/jf030177n

[bib80] Sayin SI, Wahlström A, Felin J et al. Gut microbiota regulates bile acid metabolism by reducing the levels of tauro-beta-muricholic acid, a naturally occurring FXR antagonist. Cell Metab. 2013;17:225–35. 23395169 10.1016/j.cmet.2013.01.003

[bib81] Schwartz AJ, Das NK, Ramakrishnan SK et al. Hepatic hepcidin/intestinal HIF-2α axis maintains iron absorption during iron deficiency and overload. J Clin Invest. 2019;129:336–48. 30352047 10.1172/JCI122359 PMC6307944

[bib82] Shanmugam NKN, Chen K, Cherayil BJ. Commensal bacteria-induced interleukin 1β (IL-1β) secreted by macrophages up-regulates hepcidin expression in hepatocytes by activating the bone morphogenetic protein signaling pathway. J Biol Chem. 2015;290:30637–47. 26515063 10.1074/jbc.M115.689190 PMC4683283

[bib83] Shen J, Sheng X, Chang Z et al. Iron metabolism regulates p53 signaling through direct heme-p53 interaction and modulation of p53 localization, stability, and function. Cell Rep. 2014;7:180–93. 24685134 10.1016/j.celrep.2014.02.042 PMC4219651

[bib84] Shen Y, Li X, Su Y et al. HAMP downregulation contributes to aggressive hepatocellular carcinoma via mechanism mediated by cyclin4-dependent kinase-1/STAT3 pathway. Diagnostics. 2019;9:48. 31052210 10.3390/diagnostics9020048 PMC6628061

[bib85] Shi J, Zhao Q, Hao DD et al. Gut microbiota profiling revealed the regulating effects of salidroside on iron metabolism in diabetic mice. Front Endocrinol. 2022;13:1014577. 10.3389/fendo.2022.1014577 PMC9539846 36213297

[bib86] Simcox JA, Mcclain DA. Iron and diabetes risk. Cell Metab. 2013;17:329–41. 23473030 10.1016/j.cmet.2013.02.007 PMC3648340

[bib87] Smith SGJ, Mahon V, Lambert MA et al. A molecular Swiss army knife: ompA structure, function and expression. FEMS Microbiol Lett. 2007;273:1–11. 17559395 10.1111/j.1574-6968.2007.00778.x

[bib88] Sousa L, Oliveira MM, Pessôa MTC et al. Iron overload: effects on cellular biochemistry. Clin Chim Acta. 2020;504:180–9. 31790701 10.1016/j.cca.2019.11.029

[bib89] Sow FB, Florence WC, Satoskar AR et al. Expression and localization of hepcidin in macrophages: a role in host defense against tuberculosis. J Leukocyte Biol. 2007;82:934–45. 17609338 10.1189/jlb.0407216

[bib90] Tsuchida T, Friedman SL. Mechanisms of hepatic stellate cell activation. Nat Rev Gastroenterol Hepatol. 2017;14:397–411. 28487545 10.1038/nrgastro.2017.38

[bib91] Udali S, Castagna A, Corbella M et al. Hepcidin and DNA promoter methylation in hepatocellular carcinoma. Eur J Clin Invest. 2018;48:e12870. 29235098 10.1111/eci.12870

[bib92] Valenti L, Fracanzani AL, Bugianesi E et al. HFE genotype, parenchymal iron accumulation, and liver fibrosis in patients with nonalcoholic fatty liver disease. Gastroenterology. 2010;138:905–12. 19931264 10.1053/j.gastro.2009.11.013

[bib93] Vela D . Low hepcidin in liver fibrosis and cirrhosis; a tale of progressive disorder and a case for a new biochemical marker. Mol Med. 2018;24:5. 30134796 10.1186/s10020-018-0008-7 PMC6016890

[bib94] Wan Z, Zheng J, Zhu Z et al. Intermediate role of gut microbiota in vitamin B nutrition and its influences on human health. Front Nutr. 2022;9:1031502. 36583209 10.3389/fnut.2022.1031502 PMC9792504

[bib95] Wang C-Y, Babitt JL. Hepcidin regulation in the anemia of inflammation. Curr Opin Hematol. 2016;23:189. 26886082 10.1097/MOH.0000000000000236 PMC4993159

[bib96] Wang H, Li H, Jiang X et al. Hepcidin is directly regulated by insulin and plays an important role in iron overload in streptozotocin-induced diabetic rats. Diabetes. 2014;63:1506–18. 24379355 10.2337/db13-1195

[bib97] Wang T, Zhang K-H. New blood biomarkers for the diagnosis of AFP-negative hepatocellular carcinoma. Front Oncol. 2020;10:1316. 32923383 10.3389/fonc.2020.01316 PMC7456927

[bib98] Wang X-Q, Zhang A-H, Miao J-H et al. Gut microbiota as important modulator of metabolism in health and disease. RSC Adv. 2018;8:42380–9. 35558413 10.1039/c8ra08094a PMC9092240

[bib99] Weiskirchen R, Weiskirchen S, Tacke F. Recent advances in understanding liver fibrosis: bridging basic science and individualized treatment concepts. F1000Res. 2018;7:1–17. 10.12688/f1000research.14841.1 PMC6024236 30002817

[bib100] Wieland A, Frank DN, Harnke B et al. Systematic review: microbial dysbiosis and nonalcoholic fatty liver disease. Aliment Pharmacol Ther. 2015;42:1051–63. 26304302 10.1111/apt.13376

[bib101] Xia Y, Babitt JL, Sidis Y et al. Hemojuvelin regulates hepcidin expression via a selective subset of BMP ligands and receptors independently of neogenin. Blood, The Journal of the American Society of Hematology. 2008;111:5195–204. 10.1182/blood-2007-09-111567 PMC2384142 18326817

[bib102] Xiao X, Alfaro-Magallanes VM, Babitt JL. Bone morphogenic proteins in iron homeostasis. Bone. 2020;138:115495. 32585319 10.1016/j.bone.2020.115495 PMC7453787

[bib103] Xiong X-P, Zhang BW, Yang MJ et al. Identification of vaccine candidates from differentially expressed outer membrane proteins of Vibrio alginolyticus in response to NaCl and iron limitation. Fish Shellfish Immunol. 2010;29:810–6. 20659563 10.1016/j.fsi.2010.07.027

[bib104] Yilmaz B, Li H. Gut microbiota and iron: the crucial actors in health and disease. Pharmaceuticals. 2018;11:98. 30301142 10.3390/ph11040098 PMC6315993

[bib105] Younossi ZM, Stepanova M, Younossi Y et al. Epidemiology of chronic liver diseases in the USA in the past three decades. Gut. 2020;69:564–8. 31366455 10.1136/gutjnl-2019-318813

[bib106] Zhang S, Zhao J, Xie F et al. Dietary fiber-derived short-chain fatty acids: a potential therapeutic target to alleviate obesity-related nonalcoholic fatty liver disease. Obes Rev. 2021;22:e13316. 34279051 10.1111/obr.13316

[bib107] Zheng Z, Wang B. The gut-liver axis in health and disease: the role of gut microbiota-derived signals in liver injury and regeneration. Front Immunol. 2021;12:1–14. 10.3389/fimmu.2021.775526 PMC8703161 34956204

[bib108] Zmijewski E . TLR4 signaling and the inhibition of liver hepcidin expression by alcohol. World J Gastroenterol. 2014;20:12161. 25232250 10.3748/wjg.v20.i34.12161 PMC4161801

